# Insight into picophytoplankton diversity of the subarctic White Sea—The first recording of Pedinophyceae in environmental DNA

**DOI:** 10.1002/mbo3.892

**Published:** 2019-06-11

**Authors:** Irina A. Milyutina, Tatyana A. Belevich, Lyudmila V. Ilyash, Aleksey V. Troitsky

**Affiliations:** ^1^ Belozersky Institute of Physico‐Chemical Biology Lomonosov Moscow State University Moscow Russia; ^2^ Biological Faculty Lomonosov Moscow State University Moscow Russia

**Keywords:** environmental DNA, Marsupiomonadales, Pedinophyceae, picophytoplankton, rDNA sequence, rRNA secondary structure, White Sea

## Abstract

Operational taxonomic units 94%–95% similar to the known Pedinophyceae were found as a result of high‐through sequencing of 18S rDNA V4 amplicons of environmental DNA from the summer picophytoplankton samples from the White Sea. Partial sequence of a ribosomal operon (the 5,298 bp includes partial 18S and 28S rDNA, complete 5.8S rDNA, ITS1, and ITS2 sequences) and a partial 2,112 bp chloroplast 23S rDNA sequence White Sea Pedinophyceae was amplified from metagenomic DNA by specific primers and sequenced. A new phylotype was designated as uncultured Pedinophyceae WS. On Chlorophyta phylogenetic trees the discovered phylotype occupies a basal position in the Marsupiomonadales clade. The synapomorphic base substitutions in rRNA hairpins confirm the relationship of Pedinophyceae WS to Marsupiomonadales and its difference from known genera of the order. The obtained results extend knowledge of picophytoplankton diversity in subarctic waters.

## Graphical abstract

Operational taxonomic units 94%–95% similar to the known Pedinophyceae were found as a result of high‐through sequencing of 18S rDNA V4 amplicons of environmental DNA from the summer picoplankton samples from the White Sea. Partial sequence of a ribosomal operon (the 5,298 bp includes partial 18S and 28S rDNA, complete 5.8S rDNA, ITS1, and ITS2 sequences) and a partial 2,112 bp chloroplast 23S rDNA sequence White Sea Pedinophyceae was amplified from metagenomic DNA by specific primers and sequenced. A new phylotype was designated as uncultured Pedinophyceae WS. On Chlorophyta phylogenetic trees the discovered phylotype occupies a basal position in the Marsupiomonadales clade. The synapomorphic base substitutions in rRNA hairpins confirm the relationship of Pedinophyceae WS to Marsupiomonadales and its difference from known genera of the order. The obtained results extend knowledge of picophytoplankton diversity in subarctic waters.

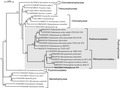

## 1 INTRODUCTION

Over half of global photosynthetic production occurs in the oceans, with pico‐sized cyanobacteria and eukaryotes (0.8–3 μm diameter) frequently accounting for much of this production (Forest et al., 2011; Jardillier, Zubkov, Pearman, & Scanlan, 2010). Photosynthetic picoeukaryotes, although less abundant in numbers than cyanobacteria, can dominate in terms of standing carbon stocks (Li, 1994; Worden, Nolan, & Palenik, 2004), and they are phylogenetically diverse with the presence of many uncultured lineages (Vaulot, Eikrem, Viprey, & Moreau, 2008). Among the picoforms there are representatives of the Pedinophyceae—a small class of green flagellates with a single emergent flagellum, which was established by Moestrup (1991).

According to recent taxonomic revision (Marin, 2012; Wang, Lin, Goes, & Lin, 2016), a classification of the Pedinophyceae comprises of two orders (Pedinomonadales and Marsupiomonadales), three families (Pedinomonadaceae, Marsupiomonadaceae, and Resultomonadaceae), and four genera (*Pedinomonas*, *Marsupiomonas*, *Protoeuglena,* and *Resultomonas*).

It is believed that only the order of Marsupiomonadales comprises marine and brackish water species, whereas Pedinomonadales includes freshwater and soil species (Marin, 2012). Today one species of Marsupiomonadaceae (*Marsupiomonas pelliculata* Jones, Leadbeater et Green) and one species of Resultomonadaceae (*Resultomonas moestrupii* Marin, invalid synonyms *Resultor mikron* (Throndsen) Moestrup, *Pedinomonas mikron* Throndsen) are only described. The information about the biogeography of Marsupiomonadales is quite scarce. One reason for the lack of information is probably a pico‐size of Marsupiomonadales, and their small size mostly hinders microscopic identification on lower taxonomic levels (Vaulot et al., 2008). Using light and in some cases electron microscopy, *Resultomonas moestrupii* has been reported previously as *Resultor mikron * from Denmark and Australia (Moestrup, 1991), Japan (Moestrup, 1992, cited by Thomsen & Buck, 1998), the California coast (Thomsen & Buck, 1998), the Norwegian Sea (Bratbak et al., 2011; Moestrup, 1991), and the Kara Sea (Sukhanova, Flint, Sazhin, Sergeeva, & Druzhkova, 2015). As for *Marsupiomonas pelliculata*, the cells of this picoalga were initially isolated from samples collected in a salt marsh in the Tamar Estuary, Cornwall (Jones, Leadbeater, & Green, 1994).

Precise taxonomical identification and the accurate phylogenetic affiliation of pico‐sized Pedinophyceae require the use of molecular methods. Three incomplete sequences of 18S rDNA uncultured eukaryotes deposited in the NCBI GenBank were referred to as Marsupiomonadales by BLAST analysis. The first one is a eukaryote that was found in a sample collected at Long Island, New York (FJ221481). The second uncultured eukaryote (KC879111) from the order of Marsupiomonadales has detected in winter ice‐covered picoplankton from the alkaline Zab‐szék shallow pan in Hungary (Pálffy et al., 2014). The third eukaryote (KC539447) was discovered in the Dapeng Bay, Taiwan in a coastal lagoon (Kuo et al., 2014).

Most molecular phylogeny studies of Pedinophyceae used the sequences of nuclear and plastid ribosomal genes (Marin, 2012; Sym, 2015; Wang et al., 2016). The data of the nuclear ribosomal genes of Marsupiomonadales in the GeneBank are represented mainly by the 18S rRNA sequences. They are four sequences of the *Marsupiomonas* 18S rRNA gene, three of which are *Marsupiomonas pelliculata*. Besides, in the GenBank there are 18S rDNA sequences of 16 clones of *Pedinomonas noctilucae* (Subrahmanyan) Sweeney*,* endosymbiotic algae of dinoflagellate *Noctiluca scintillans*. As shown by Wang et al. (2016), *Pedinomonas noctilucae* forms a monophyletic clade sister to *Marsupiomonas pelliculata* necessitating the placement of the endosymbiotic algae as an independent genus within the family Marsupiomonadaceae. It has been proposed to reinstate the genus *Protoeuglena* and reclassify the endosymbiont as *Protoeuglena noctilucae* R.Subrahmanyan (Wang et al., 2016). The GenBank also contains a single complete sequence of the 5.8S rDNA and the incomplete sequence of 28S rDNA of *Marsupiomonas pelliculata*, the whole chloroplast genome *Marsupiomonas* sp. NIES 1824, as well as incomplete sequences of chloroplast 16S and 23S rDNA of *Marsupiomonas pelliculata* and *Resultomonas moestrupii*. There are no sequences of nuclear rDNA from *Resultomonas* in the GenBank. Thus, at present, few species of the Marsupiomonadales are known.

The White Sea, a marginal subpolar shelf region basin adjoins the Barents Sea to the south of the Kola Peninsula and has features similar to those of the Arctic shelf seas (Berger et al., 2001). The sea is usually covered with ice for 5–6 months, from December to May. In summer, the temperature of the surface layer is similar to that in temperate waters. Phytoplankton of the White Sea is studied in detail during last 30 years (Ilyash, Belevich, Zhitina, Radchenko, & Ratkova, 2018). The species composition of nano‐ and microphytoplankton has been studied by microscopy; the list of algae contains 449 species. The diversity of picophytoplankton has not been extensively studied, in part because of their small size and lack distinctive morphological features. Previous studies of picoforms taxonomic diversity in plankton and ice of the White Sea by next generation sequencing (NGS) of the 18S rDNA V4 region revealed phototrophic picoeukaryotes from eight algae classes Mamiellophyceae, Pyramimonadophyceae, Palmophyllophyceae, Trebouxiophyceae, Bolidophyceae, Pelagophyceae, Mediophyceae, and Coccolithophyceae (Belevich et al., 2015, 2018; Belevich, Ilyash, Milyutina, Logacheva, & Troitsky, 2017a, 2017b). Besides NGS studies have detected sequences with 94%–95% similarity to Marsupiomonadales by BLAST analysis. To confirm the presence of Pedinophyceae in the waters of the White Sea and to understand their position within Marsupiomonadales we synthesized specific primers complementary to the V4 variable region to amplify and sequence 5,298 bp of a nuclear ribosomal operon of the uncultured Pedinophyceae from environmental DNA (eDNA) sample from the White Sea. Primers specific to the chloroplast 23S rRNA gene were synthesized on the basis of Chlorophyta 23S rDNA alignment including data for known Marsupiomonadales (*Marsupiomonas* and *Resultomonas*) and used to amplify and sequence a fragment of the 23S rRNA gene of the uncultured Pedinophyceae from the same DNA preparations. Analysis of phylogenetic trees and rRNAs secondary structure allows us to refer a discovered Pedinophyceae phylotype to Marsupiomonadales and designate it as Pedinophyceae WS.

## 2 MATERIALS AND METHODS

### 2.1 Sample collection

**Figure A1:**
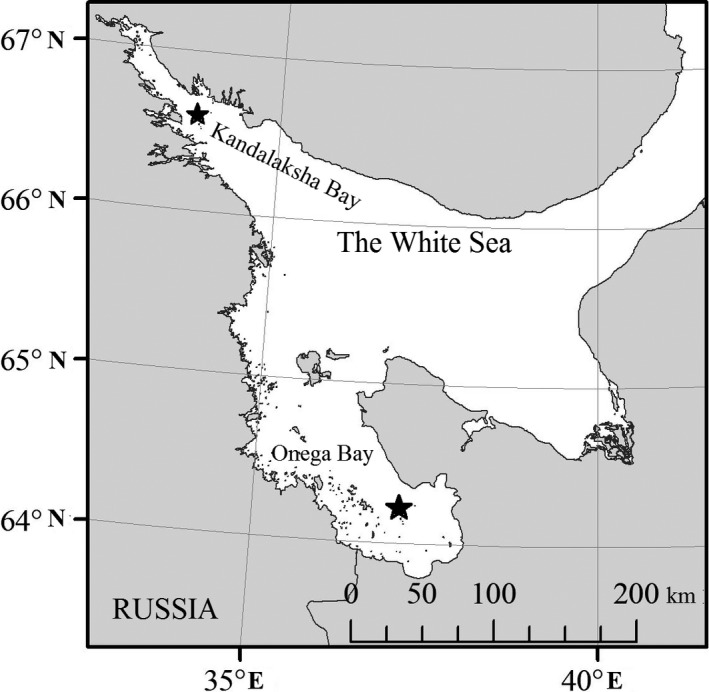
Map of sampling sites

The sampling was carried out in Kandalaksha Bay in July 2014 in the vicinity of the White Sea Biological Station, Lomonosov Moscow State University (66° 33′N, 33° 06′E) and in June 2015 by the research vessel Ekolog in Onega Bay. Locations of the sampling stations and environmental parameters are presented in Table 1 and at Figure A1. In the Kandalaksha Bay samples were collected in the surface layer (0 m) and in the Onega Bay in the layer of chlorophyll maximum (4 m). At each station, temperature and salinity were measured. In Kandalaksha Bay in July 2014, the salinity was measured with a conductivity meter Cond 3150i (WTW, Germany) and temperature—by Testo 108 (Testo, Germany). In Onega Bay in June 2015, the vertical sounding of the water column was performed with the hydrological probes CastAway (SonTek) and SBE25 (SeaBird Scientific) to measure water temperature and salinity. Water samples were taken with a Niskin bottle (5 L), and then 2–3 L of water sample was passed through an inverse filtering chamber (filter pore diameter, 3 μm) to remove nano‐ and microplankton. The filtrate (<3 μm) was then filtered through 0.2 μm Sterivex units (Millipore Canada Ltd., Canada). To the Sterivex units 1.8 ml of 50 mM Tris–HCl, 0.75 M sucrose, and 40 mM EDTA, pH 8.3 (Potvin & Lovejoy, 2009) was added, and the samples were stored at −80°C until nucleic acid extraction.

**Table 1:** Location of the sampling stations in the White Sea and environmental parameters in July 2014 and June 2015

Sample	Latitude(^o^N) Longitude(^o^E)	Date	Depth,m	Temperature, °C	Salinity, psu
Kandalaksha Bay	66^o ^32.01′ 33^o^6.54′	July 20, 2014	0	15	24.5
Onega Bay	64°21.04′ 37°02.85′	June 24, 2015	4	11	24.2

### 2.2 DNA isolation, amplification, and sequencing

eDNA was isolated using a NucleoSpin Plant kit (Macherey‐Nagel, Germany).To amplify the uncultured Pedinophyceae rDNA, we used nested‐PCR.

The primers were designed as a result of visual examination of the alignment of the 18S rDNA sequences of different Chlorophyta, including all available Pedinophyceae from GenBank, and operational taxonomic units of the uncultured Pedinophyceae WS found by NGS sequencing. The alignment was performed by MAFFT 7.4.09 (Katoh & Standley, 2013).

Primers complementary to V4 variable regions of the 18S rRNA gene and containing nucleotides substitutions at the 3′‐ends, unique to Pedinophyceae WS were synthesized. These are a pair of forward primers—Pdir1 and Pdir2 and reverse primer Prev2 (Table 2).

**Table 2:** Amplification primers

PCR primer	Sequence (5′ → 3′)
Pdir1^a^	GATTTCGGGCGGGTTCCA
Pdir2^a^	GATCGGGCTTCGGTTCGAG
Prev2^a^	CTCGCGGAACTCGAACCGAAG
Pdir3^a^	CCTCAGCCTGCTAAATAGCTAC
Pdir4^a^	GACTTTCGGGGTTTTACCCGGA
A^b^	CCTGGTTGATCCTGCCAGT
NLR204^c^	ATATGCTTAARTTCAGCGGGT
prITS2(rev)^d^	GCTGCGTTCTTCATCGATGC
NLR3535^c^	MRGGCTKAATCTCARYRGATCG
NLR3284^c^	TTCTGACTTAGAGGCGTTCAG
23dir1^a^	CGGTGGATACCTAGGCATTC
23rev3^a^	TAGCTACCCAGCGTTTCCC
23dir2^a^	CGCGAGGGAAAGGTGAAAGAG
23rev1c^a^	GACCGAACTGTCTCACGACG

^a^ Primers constructed in this study.

^b^ Medlin, Elwood, Stickel, and Sogin (1988).

^c^ Data base “Primers for Eukaryotic Nuclear LSU rRNA” (http://bio.cug.edu.cn/rRNAprimers/NL_lst.html).

^d^ White, Bruns, Lee, and Taylor (1990).

To amplify the portion of the ribosomal operon to the 3′‐end of the V4 region, direct primers Pdir1 (in the first round of PCR) and Pdir2 (in the second round) were used. Reverse primers complementary to conserved regions of ribosomal genes were used: prITS2—complementary to the 5.8S rRNA gene and NLR204—complementary to the 5′‐end of the 28S rRNA gene (Table 2).

To sequence the part of the 18S rRNA gene between the 5′‐end and the V4 region, amplicons were obtained using forward primer A complementary to the 5′‐end of the 18S rRNA gene and reverse Prev2 primer specific to the Pedinophyceae WS. Thus, the sequences of 18S rRNA gene, ITS1, the 5.8S rRNA gene, the ITS2, and the 5′‐end of the 28SrRNA gene were determined.

The resulting 18S rDNA sequence of Pedinophyceae WS was aligned with the known 18S rDNA of Pedinophyceae from GenBank. To amplify the 28S rRNA gene, we synthesized a second pair of forward primers complementary to V7 variable region of the 18S rRNA gene. The primers are Pdir3 (first PCR round) and Pdir4 (second round). They also have unique substitutions at the 3′‐ends, specific only for the uncultured Pedinophyceae WS.

With this pair of primers and reverse primers complementary to the conserved regions of the 28S rRNA gene (NLR3535 in the first PCR round and NLR3284in the second round), an amplicon of about 4.2 kb, containing the incomplete 28S rRNA gene sequence (3,042 bp), was obtained. It was sequenced using a series of primers complementary to the conserved regions of the 28S rRNA gene (Table 3).

**Table 3:** Sequencing primers

PCR primer	Sequence (5′ → 3′)
prITS3(dir)^a^	GCATCGATGAAGAACGCAGC
NLF184^b^ (nuclear LSU rRNA)	ACCCGCTGAAYTTAAGCATAT
NLF796^b^ (nuclear LSU rRNA)	GTCTTGAAACACGGACCAAGG
NLF1410^b^ (nuclear LSU rRNA)	TCCGCTAAGGAGTGTGTAACAAC
NLF2075^b^ (nuclear LSU rRNA)	GTCACTTCGGGAWAAGGATTGGCT
23rev2c^c^	TGCCGAGTTCCTTAGAGAGAGT

^a^ White et al. (1990).

^b^ Data base “Primers for Eukaryotic Nuclear LSU rRNA” (http://bio.cug.edu.cn/rRNAprimers/NL_lst.html).

^c^ Primer constructed in this work.

The 23S chloroplast rDNA region of uncultured Pedinophyceae WS was amplified from the same Kandalaksha Bay sample, in which nuclear rRNA genes were discovered. For the amplification primers with unique substitutions at the 3′‐end, which are characteristic of available in the GenBank sequences of the chloroplast 23S rDNA of *Marsupiomonas* and *Resultomonas*, were synthesized. Primers of the first round of PCR are 23dir1 and 23rev3 and of the second round are 23dir2 and 23rev1c (Table 2). To sequence the amplified fragment, the internal primer 23rev2c, complementary to the conserved region of the gene, was also used (Table 3). The length of the partial sequence of the chloroplast 23S rRNA gene was 2,112 bp. PCR products of both nuclear and chloroplastic ribosomal genes in agarose gel electrophoresis moved as single bands.

PCR was performed in a 25 µl reaction mix. Cycling conditions were as follows: initial denaturation at 95°C for 3 min; in each cycle, denaturation was carried out for 20 s at 95°C. The annealing temperature was determined by the primer melting temperature, with the annealing time of 20 s. Elongation run was 1 min. per each 1,000 nucleotides at 72°C. A final extension at 72°C was carried out for 5–10 min. The cycle number was 25 in the first PCR round and 35 in the second. The DNA matrix used in the second PCR round was 1 µl PCR‐product of the first round. The DNA was amplified using a ready mix for PCR—ScreenMix‐HS (Evrogen, Russia), containing high‐processivity Taq‐DNA Polymerase without corrective 3′ > 5′ exconuclease activity.

After preparative electrophoresis in 1% agarose gel, PCR products were cut out of the gel and purified with the Cleanup Mini kit (Evrogen, Russia). DNA sequencing was carried out using a set of reagents ABI PRISM® BigDye Terminator v. 3.1, followed by analysis of the reaction products on the automatic sequencer Applied Biosystems 3,730 DNA Analyzer (Life Technologies).

The sequences have been deposited in GenBank under accession numbers MK030604, and MK550895 (nuclear rDNA) and MK030605 (23S chloroplast rDNA).

### 2.3 Phylogenetic and rRNA secondary structure analysis

Three data sets were used for phylogenetic trees reconstruction: nuclear‐encoded 18S rDNA, 18S + 5.8S + 28S rDNA, and chloroplast 23S rDNA. Alignments of nucleotide sequences were performed by MAFFT‐7.4.09 software (Katoh & Standley, 2013) and adjusted by eye. Intron sequences, intergenic spacers, and ambiguously aligned regions were excluded. The final alignment lengths were 1,806 bp for 18S rDNA, 5,103 bp for 18S + 5.8S + 28S rDNA, and 2,266 bp for 23S rDNA. The phylogenetic trees were inferred by the maximum likelihood method using RAxML 8.2.10 program (Stamatakis, 2014) with default options. The bootstrap replicates numbers were set by bootstrapping criterion implemented in RAxML.

**Figure A2:**
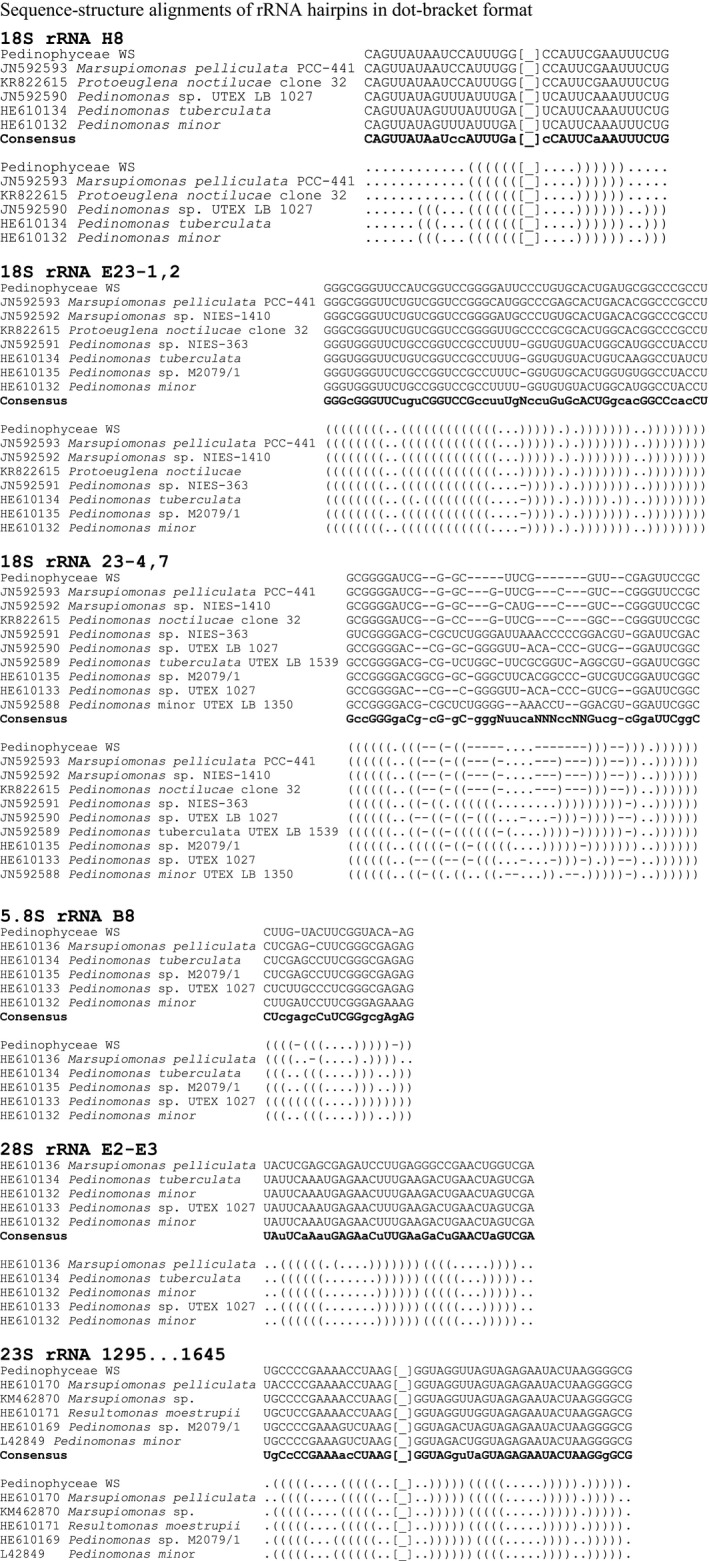
Sequence‐structure alignments of hairpins in dot‐bracket format

The secondary structures of the terminal hairpins of V4 rRNA region were predicted by mfold (Zuker, 2003). Internal hairpins were constructed according eubacterial 23S rRNA general model (Gutell & Fox, 1988) or small subunit RNA secondary structure model (Wuyts et al., 2000). Hairpins were drawn by RnaViz 2.0.3 program (De Rijk, Wuyts, & Wachter, 2003). Sequence‐structure alignments in dot‐bracket format computed on the MARNA server (Raden et al., 2018) are shown in the Figure A2.

## 3 RESULTS

### 3.1 Sequence analysis

**Figure A3:**
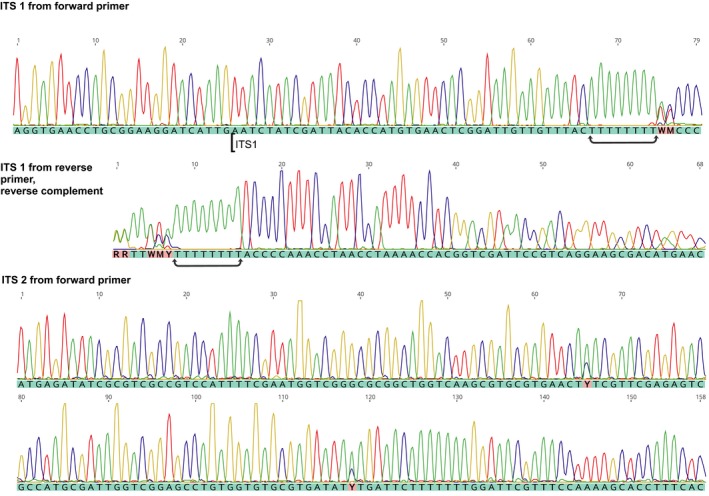
Sanger electrophoregram of ITS 1 and ITS 2 regions of Pedinophyceae WS DNA with indication of polymorphic sites

We sequenced a 5,298 bp portion of a ribosomal operon of the Pedinophyceae WS from Kandalaksha Bay, including the genes 18S, 5.8S, and 28S rRNA and the spacers ITS1 and ITS2, and 2,112 bp of the chloroplast 23S rRNA gene. We then sequenced 2,312 bp of a ribosomal operon of the Pedinophyceae WS from Onega Bay (18S, 28S rDNA partial sequences, and 5.8S rDNA, ITS1, and ITS2 complete sequences). Only minor heterogeneity in Sanger electrophoregram was detected: two polymorphic sites (C/T) in ITS 2 and the presence of 8 or 9 bp in oligo‐T block in ITS 1 (Figure A3). Sequences from both locations are identical. No other sequences corresponding to Pedinophyceae were detected in the samples by BLAST analysis of the NGS results. It means that only one taxon of Pedinophyceae presents in two studied plankton samples of the White Sea. Sequence similarities between rDNA of the Pedinophyceae WS and other known pedinophytes are shown in Table 4.

**Table 4:** The similarity of rDNA sequences of uncultured Pedinophyceae WS with other Pedinophyceae (in %)

Species, phylotype	18S rDNA	5.8S rDNA	28S rDNA	23S rDNA
*Marsupiomonas pelliculata*	94.5 FR865498	89.4 FR865498	92.0 HE610137	91.4 HE610170
*Marsupiomonas* sp. NIES‐1410	95.2 JN592592	No data	No data	92.7 KM462870
*Pedinomonas noctilucae* clone 2	94.2 KR822604	No data	No data	No data
*Resultomonas moestrupii*	No data	No data	No data	87.8 HE610171
*Pedinomonas minor*	89.7 HE610132	86.5 HE610132	84.7 HE610132	87.8 NC016733
*Pedinomonas tuberculata*	89.8 HE610134	84.5 HE610134	84.9 HE610134	87.2 KM462867
*Pedinomonas* sp. M2079/1 HE610135	89.2 HE610135	84.5 HE610135	85.3 HE610135	87.2 HE610169
*Pedinomonas* sp. UTEX 1027	89.5 HE610133	87.1 HE610133	85.4 HE610133	87.2 HE610167
*Pedinomonas* sp. NIES‐363	90.4 JN592591	No data	No data	No data

Note

The GenBank accession numbers of compared sequences are indicated along with similarity values.

The length of the ITS1 of the Pedinophyceae WS is only 105 bp, which is about two times shorter than that of the Pedinomonadales species (HE610134, HE610132). The order of Marsupiomonadales in the GenBank is represented by a single incomplete sequence of ITS1 *Marsupiomonas pelliculata* (FR865498). The 5′‐ and 3′‐end regions of the ITS1 sequence are conserved in all known pedinophytes, including the Pedinophyceae WS. The length of ITS2 of the Pedinophyceae WS is 226 bp. There are no Marsupiomonadales ITS2 sequences in the GenBank.

### 3.2 Phylogenetic trees reconstruction

**Figure 1:**
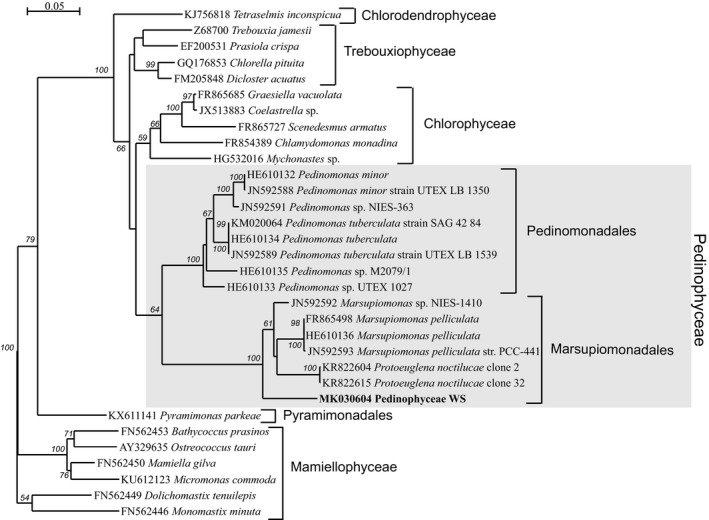
Maximum likelihood phylogenetic tree based on nuclear 18S rDNA sequences from 32 Chlorophyta taxa. Bootstrap supports > 50% are indicated at the nodes

The phylogenetic tree of nuclear‐encoded 18S rRNA from 32 Chlorophyta taxa with 15 Pedinophyceae phylotypes is shown in Figure 1. The uncultured Pedinophyceae WS is embedded in Marsupiomonadales clade where it occupies a basal position. The mean similarity percentages of Pedinophyceae 18S rRNA are indicated in Table 5.

**Table 5:** The mean similarity (in %) of Pedinophyceae 18S rDNA

	Pedinophyceae WS	*Protoeuglena*	*Marsupiomonas*
*Protoeuglena*	94.6		
*Marsupiomonas*	95.5	93	
*Pedinomonas*	91	91.5	91.6

**Figure 2:**
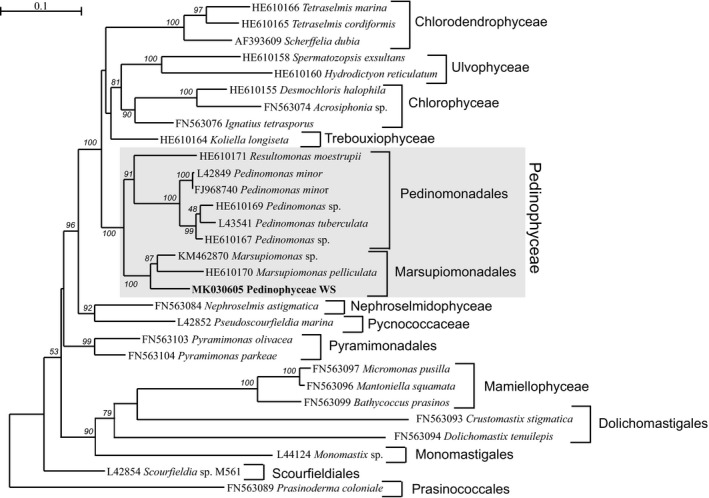
Maximum likelihood phylogenetic tree based on plastid 23S rDNA sequences from 30 Chlorophyta taxa. Bootstrap supports > 50% are indicated at the nodes

Available data for the nuclear‐encoded rRNA operon are more limited and includes only four *Pedinomonas* and one *Marsupiomonas* taxa. In the phylogenetic tree for these sequences, Pedinophyceae WS clustered with *M.  pelliculata* with maximum bootstrap support in a clade sister to the clade of *Pedinomonas* (data not shown). On the chloroplast 23S rDNA tree (Figure 2), this taxon is a sister to *Marsupiomonas* species as well.

### 3.3 18S rRNA secondary structure analysis

**Figure 3:**
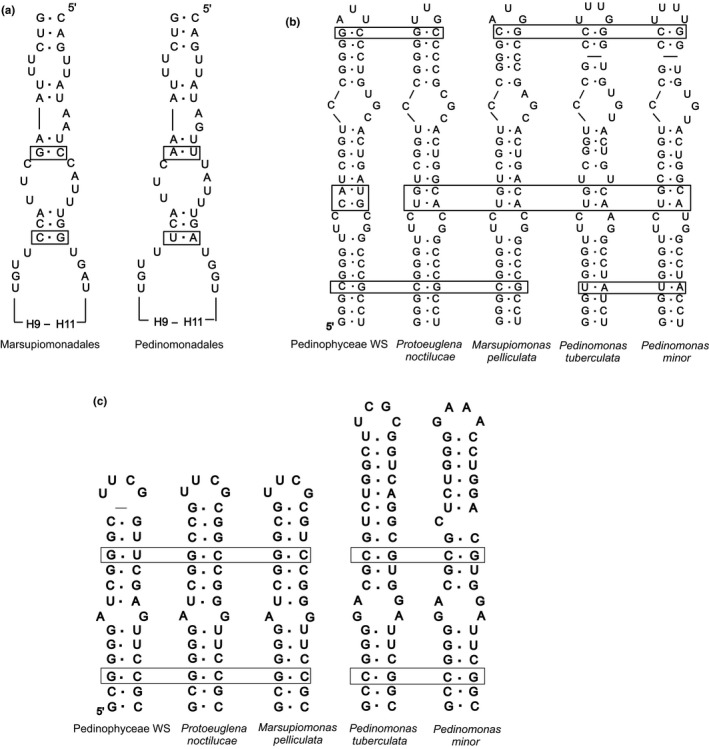
The secondary structure of 18S rRNA hairpins from Pedinophyceae. (a) H8 hairpin, (b) E23‐1,2 hairpins (c) E23‐4,7 hairpins

In the secondary structure of Pedinophyceae 18S rRNA, there is a number of compensatory base changes (CBC) differentiating the Marsupiomonadales (*Marsupiomonas* and *Protoeuglena noctilucae*) from the Pedinomonadales (*Pedinomonas minor* and *P.  tuberculata*). Thus, hairpin H8 (hairpin numbering is given according to the model of the secondary structure of the red algae *Palmaria palmata* 18S rRNA, Wuyts et al., 2000) in Marsupiomonadales, including the Pedinophyceae WS, has two CBC which are different from those in Pedinomonadales—CG > UA and GC > AU (Figure 3a). A number of CBC in V4 variable region hairpins of the 18S rRNA confirms the affinity of Pedinophyceae WS to Marsupiomonadales.

So, the apical part of the hairpin E23‐1,2 from Pedinomonadales has no paired G‐C found in *Marsupiomonas*, *P.  noctilucae*, and Pedinophyceae WS (Figure 3b). In the hairpin E23‐1, the fourth nucleotide pair in the Pedinomonadales is U‐A, whereas in the known of Marsupiomonadales and Pedinophyceae WS it is C‐G.

There are apparent differences between E23‐4,7, another pair of hairpins of the V4 variable region, in Marsupiomonadales and Pedinomonadales (Figure 3c). The apical part of the hairpin E23‐7 in Marsupiomonadales is shorter than in Pedinomonadales due to deletion of a number of nucleotides, and the same features are found in this hairpin in Pedinophyceae WS. In the conservative basal part of the E23‐4 hairpin, the third pair in Pedinomonadales is C‐G, whereas in *M.  pelliculata*, *P.  noctilucae,* and Pedinophyceae WS it is G‐C. The second CBC is in the fourth pair of hairpins E23‐7; C‐G in Pedinomonadales is changed to G‐Y in known Marsupiomonadales and Pedinophyceae WS (Figure 3c).

At the same time, the structure of these hairpins in Pedinophyceae WS, has unique features that distinguish it from other Marsupiomonadales, in particular, two CBC in the hairpin E23‐2, UA > CG and GC > AU, in the first and second pairs of the hairpin. Pedinophyceae WS, unlike other taxa of the order, does not have a pair of nucleotides G‐C in the apical part of hairpin E23‐7.

### 3.4 5.8S rRNA secondary structure analysis

**Figure 4:**
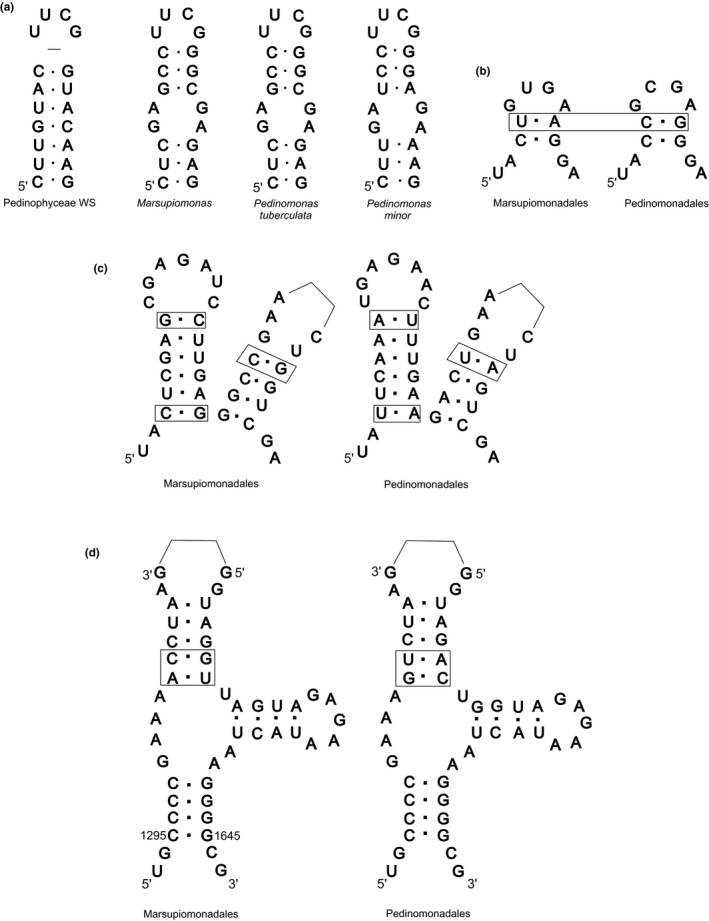
The secondary structure of rRNAs hairpins from Pedinophyceae. (a) B8 hairpin of 5.8S rRNAs, (b) B20 hairpins of 28S rRNAs, (c) hairpins E2 and E3 of 28S rRNAs, (d) hairpins of 23S rRNAs (position numbers for 23S rRNA *Escherichia coli*)

3′‐end of 5.8 S rRNA Pedinophyceae WS, which forms a hairpin with 5′‐end of 28S rRNA in the secondary structure, is highly conserved and has no substitutions, like other Pedinophyceae. The hairpin B8 (numbered according to De Rijk et al., 1999) preceding the 3′‐end hairpin in Pedinophyceae WS has a number of features distinguishing it from other Pedinophyceae (Figure 4a). Due to the deletion of the apical pair of C‐G nucleotides, this hairpin includes seven pairs of nucleotides rather than eight like the rest of Pedinophyceae. All Pedinophyceae in the central part of the hairpin has an internal loop formed by the unpaired bases GA and AG, which are replaced in uncultured Pedinophyceae WS by complementary pairs G‐C and U‐A.

### 3.5 28S rRNA secondary structure analysis

In the GenBank, there are only five sequences of the Pedinophyceae 28S rRNA gene. Four of them belong to *Pedinomonas* and one to *Marsupiomonas pelliculata*. Pedinophyceae WS is the second taxon of Marsupiomonadales, for which this gene has been sequenced. Examination of the 28S rRNA secondary structures from known Pedinophyceae allows us to identify synapomorphic features typical of each of the two pedinophytes orders. Thus, in the short conservative hairpin B20 (numbered according to De Rijk et al., 1999), the apical C‐G pair in the Pedinomonadales is replaced with the U‐A pair in the Marsupiomonadales taxa (Figure 4b). Another area with three compensatory substitutions, which draws a borderline between the two orders, is the hairpins E2 and E3 (Figure 4c).

### 3.6 23S rRNA secondary structure analysis

The secondary structure of the chloroplast 23S rRNA gene of the Pedinophyceae WS also has features confirming its affinity to Marsupiomonadales. As an example, we use a conservative hairpin, which is formed by nucleotides 1,295–1,308 and 1,621–1,645 (Gutell & Fox, 1988) in the 23S rRNA structure of *Escherichia coli*. Two CBCs in this hairpin of Marsupiomonadales are different from that of Pedinomonadales (C‐G and A‐U in all known Marsupiomonadales, including the Pedinophyceae WS, and U‐A and G‐C in Pedinomonadales) (Figure 4d). *Resultomonas moestrupii* differs from other Marsupiomonadales by CBC in the second pair of the conservative base of the hairpin: C‐G changes to U‐A.

## 4 DISCUSSION

In the present study, a new Pedinophyceae phylotype was identified by nuclear and chloroplast rRNA sequence analysis in eDNA picoplanktonic probes from the White Sea. This finding is the first discovery of Pedinophyceae in the White Sea and the first recording of Pedinophyceae by molecular methods in the subarctic region. Discovery of a new Pedinophyceae taxon in the White Sea, the basin which combined features of a temperate waters and Arctic shelf seas (Berger et al., 2001), is relevant in light of the observed changes in marine ecosystems of the Arctic under the influence of the climate trend (McLaughlin & Carmack, 2010). In particular, there may be changes in the species composition of all size groups of phytoplankton, including picoforms, due to penetration of algae from temperate waters into the Arctic and disappearance of Arctic endemics (Lovejoy et al., 2007). Besides, the greater involvement of picoforms in primary production and more significant contribution of the smallest photoautotrophs in total phytoplankton abundance are predicted (Kilias, Wolf, Nöthig, Peeken, & Metfies, 2013; Li, McLaughlin, Lovejoy, & Carmack, 2009).

### 4.1 Affiliation Pedinophyceae WS to order of Marsupiomonadales

NGS results of the V4 region of 18S rRNA show that only one taxon of Pedinophyceae presents in two studied plankton samples of the White Sea. Therefore, we assume that the sequences of nuclear and plastid ribosomal genes belong to the same taxon of the uncultured Pedinophyceae.

The phylogenetic trees (Figures 1 and 2) show that the picoplanktonic uncultured Pedinophyceae WS is affiliated to the order of Marsupiomonadales. On the 18S rDNA tree, this taxon does not combine with either *Marsupiomonas* or *Protoeuglena*, but occupies a basal position in the Marsupiomonadales clade. On the nuclear 28S rDNA and chloroplast 23S rDNA tree, the uncultured Pedinophyceae WS is in a sister position to the genus *Marsupiomonas*. On the base of the phylogenetic trees obtained, we propose that this phylotype may correspond to the new genus of order Marsupiomonadales.

CBCs in the rRNA helixes of Pedinophyceae confirm the belonging of Pedinophyceae WS to the order of Marsupiomonadales. The partial sequence of the 28S rRNA gene from Pedinophyceae WS is the second sequence of this gene for Marsupiomonadales. A comparative analysis of the secondary structures of the Pedinophyceae ribosomal genes allowed identifying synapomorphic characters for both orders (Table 6).

**Table 6:** Compensatory base changes in rRNAs hairpins of Marsupiomonadales and Pedinomonadales taxa (Figures 3 and 4)

Hairpin numbers	Marsupiomonadales	Pedinomonadales
H8 of 18S rRNA	G‐C	A‐U
H8 of 18S rRNA	C‐G	U‐A
E23‐2 of 18S rRNA	G‐C	No
E23‐1 of 18S rRNA	C‐G	U‐A
E23‐4 of 18S rRNA	G‐C	C‐G
E23‐7 of 18S rRNA	G‐Y	C‐G
B20 of 28S rRNA	U‐A	C‐G
E2 of 28S rRNA	G‐C	A‐U
E2 of 28S rRNA	C‐G	U‐A
E3 of 28S rRNA	C‐G	U‐A
Plastid 23S rRNA	C‐G	U‐A
Plastid 23S rRNA	A‐U	G‐C

Data on the secondary structure of ribosomal genes of Pedinophyceae WS allowed clarifying some previous conclusions made regarding synapomorphies of the orders of Pedinomonadales (Marin, 2012). Thus, according to this author, the second pair in the apical part of hairpin H46 of the V8 variable region is G‐C in all Pedinomonadales; however, the G‐C pair is also observed in this position in *Protoeuglena noctilucae* and in the Pedinophyceae WS which belong to the order of Marsupiomonadales. Instead, the synapomorphic signature of the order of Pedinomonadales is the third pair on the top: for all *Pedinomonas* it is U‐A, while for *Marsupiomonas pelliculata* and *Protoeuglena noctilucae* it is C‐G, and for the Pedinophyceae WS it is A‐U.

It should be noted that the *Resultomonas moestrupii* on the 23S chloroplast DNA phylogenetic tree (Figure 2), clustered with the order of Pedinomonadales, not Marsupiomonadales, as follows from the studies of Wang et al. (2016) and Marin (2012).

### 4.2 Ecology of the new Pedinophyceae

In the White Sea the eDNA was studied in different areas (Kandalaksha and Onega bays) and various biotopes—ice, under‐ice water, and summer plankton—total 17 samples (Belevich et al., 2015, 2018, 2017a, 2017b). Pedinophyceae WS was found only in two summer plankton samples and was not found in the samples of ice and under‐ice water studied by metagenomic analysis. The water temperature in summer varied from 11 to 15°C. Other representatives of Marsupiomonadales were noted both in the under‐ice plankton temperate alkaline shallow pan at mean water temperature 0.5°C (Pálffy et al., 2014), and in the subtropical lagoon at mean water temperature 27°C (Kuo et al., 2014). In the White Sea in summer salinity varies significantly (Berger et al., 2001). In Onega Bay where Pedinophyceae WS was registered salinity varies from 8 to 27.6 psu (Belevich et al., 2016). The habitat of the Pedinophyceae WS confirms the conclusion that the order of Marsupiomonadales includes marine and brackish species (Marin, 2012).

Pedinophyceae WS was revealed in weakly stratified waters due to tidal mixing with a photic layer length of 6–26 m. There was no nutrient limitation, and the chlorophyll “a” concentration varied from 0.3 to 2 mg/m^3^ (Belevich et al., 2017b, 2016). The contribution of Pedinophyceae WS to the total number of NGS reads of photosynthetic picoeukaryotes did not exceed 1.35%. This indicates a low abundance of Pedinophyceae WS since the total amount of photosynthetic picoeukaryotes was low (0–36.9 × 10^4^ cells/L), and the molecular and microscopic signals are generally correlated (Giner et al., 2016). Similarly, the frequency of occurrence of Pedinophyceae WS is also low, since it was revealed in only two of seven metagenomic summer samples. Thus, in the summer phytoplankton of the subarctic White Sea, the Pedinophyceae WS is a rare taxon.

The Pedinophyceae WS is not the only picophytoplanktonic phylotype that was first discovered not only in the White Sea but also in subarctic waters. Our early studies revealed previously unknown phylotypes *Micromonas*, *Mantoniella,* and Bolidophyceae in the environmental DNA of the White Sea plankton (Belevich et al., 2018, 2017a). The identification of a new Pedinophyceae phylotype broadens the current understanding of the picoforms biodiversity in subarctic waters and the biogeography of this poorly studied group of photosynthetic plankton picoforms. Considering the ongoing changes in the White Sea by global warming and their implications (Pozdnyakov et al., 2007), we can expect a change in the structure of phytoplankton and, in particular, an increase of the role of rare taxa.

## CONFLICT OF INTERESTS

The authors declare no conflict of interest.

## AUTHOR CONTRIBUTIONS

IAM and TAB were involved in project administration. TAB and LVI were involved in sample collection. IAM was involved in performing the experiments. IAM, TAB, and AVT were involved in data analysis. TAB was involved in original draft preparation. IAM, TAB, LVI, and AVT were involved in review and editing.

## ETHICS STATEMENT

None required.

## Data Availability

The DNA sequences have been deposited in GenBank under accession numbers MK030604, MK550895, and MK030605.
